# Plastics and Orthopedics

**Published:** 1871-09

**Authors:** 


*Plastics and Orthopedics:* A Report re-published from the transactions of the Illinois State Medical Society for 1871. By DAVID PRINCE, M. D.

This subject has been very ably treated by the author, and exhibits a great familiarity of the author with the subject. It is illustrated with thirty-eight wood-cuts, and is a complete report of the principles, expedients and specialities of *Plastics*,, and its mechanical appliances, in the treatment of *orthopedics.*

